# Enhancement of Polysaccharides in *Ganoderma leucocontextum* by Growing with Fruit-Tree Wood–Bagasse Substrate Through Prostaglandin A1-Phosphoglucomutase Correlatively

**DOI:** 10.3390/jof12070490

**Published:** 2026-07-03

**Authors:** Yuanchao Liu, Yufan Hao, Huiping Hu, Tianqiao Yong, Manjun Cai, Huiyang Guo, Shiqi He, Xinyu Shi, Yifan Li, Zhi Zhang, Ming Jiang

**Affiliations:** 1 State Key Laboratory of Applied Microbiology Southern China, National Health Commission Science and Technology Innovation Platform for Nutrition and Safety of Microbial Food, Guangdong Provincial Key Laboratory of Microbial Safety and Health, Institute of Microbiology, Guangdong Academy of Sciences, Guangzhou 510070, China; 2 Key Laboratory of Big Data Technologies for Food Microbiological Safety, State Administration for Market Regulation, NHC Specialty Laboratory of Food Safety Risk Assessment and Standard Development, Guangdong-Hong Kong-Macao Greater Bay Area Microbiological Safety and Health International Science and Technology Innovation Center, Guangzhou 510070, China; 3 College of Life Science and Technology, Mudanjiang Normal University, Mudanjiang 157012, China; 4 Guangdong Yuewei Biotechnology Co., Ltd., Shaoguan 512029, China

**Keywords:** *Ganoderma leucocontextum*, substrate formulation, polysaccharide, metabolomics, pathway

## Abstract

*Ganoderma leucocontextum*, a fungus discovered by our group, is highly valued for its immune modulation and anti-tumor polysaccharides significantly. Thus, this research aims to select a substrate formulation to enhance its polysaccharides and reveal the underlying mechanism. Seven distinct substrate formulations, incorporating combinations of fruit-tree wood, bagasse, oak wood, and cottonseed hulls, were explored. Interesting, the fruiting bodies grown on GMTZ fruit-tree wood–bagasse formulation showed the highest polysaccharide content at 3.19 ± 0.56% (*p* < 0.01 or 0.05). Moreover, GMTZ efficiently channeled resources toward diterpenoids synthesis at the expense of flavonoid and most triterpenoid production. It also dramatically enhanced androgen synthesis, while showing no corresponding accumulation of storage lipids or certain hormone signals, reinforcing a specific metabolic commitment. Furthermore, PCA analysis of the metabolomics confirmed the profound impact of substrate formulations. Correlation analysis revealed that GMTZ promoted a growth-and-synthesis metabolic phenotype, which was characterized by metabolic signatures of supporting anabolism and cellular homeostasis. In contrast, formulations that induced the defense-and-stress phenotype were often rich in lignin, which diverted resources toward detoxification and stress responses and suppressed growth-oriented metabolite synthesis. Moreover, prostaglandin A1, deoxycholic acid, cucurbitacin E, and 1-hydroxy-2-naphthoic acid were found to be positively correlated with polysaccharide synthesis. In addition, networks for polysaccharide biosynthesis were mapped and it was proposed, accordingly, that prostaglandin A1-phosphoglucomutase may be a mechanism by which GMTZ enhances polysaccharides. This research provided a substrate formulation for elevating polysaccharides in *G. leucocontextum*.

## 1. Introduction

Edible and medicinal fungi have been used for thousands of years as traditional medicines around the world, particularly in Asia, where they are revered for their diverse health-promoting properties. This long history underscores their cultivation and therapeutic importance. Among them, *Ganoderma*, commonly known as “Lingzhi” or “Reishi,” is one of the most revered and extensively researched, with over 15,000 publications [1]. Importantly, polysaccharides (GLPs) are the main bioactive components of *G. lucidum*, with a wide range of pharmacological effects, including immunomodulatory, anti-tumor, antioxidant, anti-inflammatory, anti-fatigue, anti-hyperuricemic, hypoglycemic, hepatoprotective, and hypotensive properties [2,3,4]. These diverse health benefits of GLPs highlight their great potential as functional biomaterials, therapeutic agents, and food additives. Their wide range of pharmacological effects and huge market demand provide a high necessary for research aimed at optimizing their synthesis and production [5]. Although *G. lucidum* has been extensively studied, other *Ganoderma* species, such as *G. leucocontextum*, have also gained increasing attention due to their unique bioactivity profiles.

*G. leucocontextum*, which is found in the Tibet Plateau and is also known as white *Ganoderma* or Tibetan *Ganoderma*, was classified as a new species in 2015 [6]. *G. leucocontextum* is rich in bioactive compounds, including polysaccharides and triterpenoids, and proteins, which were 2–3 fold higher than *G. lucidum* [7] and showed anti-inflammatory, antioxidant, and anti-aging effects [8,9,10,11,12]. For instance, GLSP-A1 extracted from *G. leucocontextum* spores presented a lower molecular weight (15,750 Da) [8] than that of *G. lucidum* spore polysaccharides (81,679 Da) [13], equipping it with better bioavailability, less immune system stress, and better biological activity [14].

Nowadays, significant progress has been made in the artificial cultivation of Ganoderma species; however, achieving consistent quality and high yields of bioactive compounds remains a challenge. As such, successful cultivation is highly dependent on substrate selection, efficient strain production, and precise management of cultivation parameters [15]. As heterotrophic organisms, fungi must obtain nutrients from the external environment, mainly by degrading organic matter [16]. Therefore, the composition of the cultivation substrates directly determines the availability of carbon sources, nitrogen sources, minerals, and other micronutrients required for fungal growth and metabolism [17] and also shapes the nutrient maps of the grown fungi. Studies on *G. lucidum* and other medicinal fungi have shown that specific lignocellulosic substrates (oak, sawdust, agricultural waste) and supplements (olive oil, copper, salicylic acid, calcium ions) can significantly affect not only the yield but also the quality and active compound profiles such as triterpenes and polysaccharides [18], highlighting the importance of substrate optimization and the complexity of compound transformation in fungal growth and metabolism.

Metabolomics, the large-scale study of small molecule metabolites in biological systems, has become a revolutionary tool in fungi research [19]. It provides a snapshot of their metabolic status, revealing the dynamically metabolic profiles and target product changes in biological systems in response to environmental stimuli or genetic modifications. Unlike transcriptomics or proteomics, metabolomics can accurately reveal the direct effects of environmental perturbations or stress on fruitbodies, since these changes may be manifested at the metabolite level even if no significant change has occurred at the transcriptional or protein level [20]. In the field of edible and medicinal fungi, metabolomics has been widely used to identify quality markers, discover novel natural products, and elucidate biosynthetic pathways. For instance, it has been used to understand how sawdust addition affected the metabolism of *Hypsizygus marmoreus* mycelium, identifying amino acids, lipids, and carbohydrates as key differentially accumulated metabolites [21]. It also revealed the changing patterns of metabolites during continuous cultivation of *G. lucidum*, deepening our understanding of the impact of the soil environment on the production of *G. lucidum* metabolites and providing an important reference for optimizing *G. lucidum* growth conditions [22], especially for cultivation substrates [23]. Combining metabolomics with cultivation substrates is a novel approach to identifying which substrates work best and providing explanations of “why” and “how” specific metabolic pathways and precursor molecules are affected by the substrate. These mechanistic insights are crucial for rationally designing cultivation substrates and strategies.

However, there remains a gap in understanding how substrate formulation affects the biosynthesis and accumulation of its active polysaccharides, despite the growing interest in *G. leucocontextum*. In this study, seven formulations based on fruit-tree wood, bagasse, oak wood, and cottonseed hulls substrates were designed and their effects on total polysaccharide in *G. leucocontextume* were evaluated to select a substrate formulation for elevating polysaccharides. Furthermore, their fruiting bodies were characterized using metabolomics. Based on the metabolomic analysis, their flavone, terpenoids, and sterol nutrients were examined. In addition, their metabolism phenotype and metabolic changes were investigated using PCA. Furthermore, metabolites that were positively and negatively correlated with total polysaccharide content were annotated and mapped to KEGG maps. Finally, the metabolic pathways that may be involved in enhancing polysaccharide biosynthesis and regulation were proposed, hypothetically and correlatively. This study may provide a substrate formulation for elevating polysaccharides in *G. leucocontextum*.

## 2. Materials and Experiments

### 2.1. Strains and Substrates

Given the availability, treatment requirements of agriculture wastes, and low prices, various agro-industrial wastes were selected for preparing substrates. The seven designed culture substrates were mixed homogeneously, with a particle size of about 2 mm, moisture of 60–65% and pH of 6.5–7.0 (Table 1). The pH levels were consistent across all the formulations. The mushrooms were cultivated in polyethylene bags measuring 17 cm × 35 cm × 5 cm. Each bag was filled with 1 kg of substrates. The packing density was about 0.67 g/cm^3^. Activated *G. leucocontextum* mycelium (strain ID: I160015) of 5 mm plugs was inoculated into the bags and cultured. After the mycelium had grown and the fruiting bodies had formed, the polysaccharide content of the fruiting bodies was measured according to phehol-sulfuric acid colorimetry and the metabolites were extracted and analyzed using metabolomics to study the impacts of different substrates on *G. leucocontextum*’s development and metabolism.

### 2.2. Cultivating and Sampling G. leucocontextum

*G. leucocontextum* was cultivated under light-excluded conditions at 25 °C, 60–70% humidity, and a CO_2_ concentration at 4000 ppm for 18–20 days until mycelium filled the bag. Then, the temperature was adjusted to 26–28 °C, relative air humidity to 90–95%, and the cultures were exposed to 8 h of scattered light daily. When the primordia formed at the bag opening, the humidity was set at 80–90%. As the pileus began to differentiate, the CO_2_ concentration was lowered to under 1000 ppm. When the fruiting-body edge growth points vanished, the air humidity was adjusted to 80%. At maturity, *G. leucocontextum* was harvested.

Samples of *G. leucocontextum* bags, stipes, and pilei from different substrates were taken with tweezers and a blade. Five replicates of each sample were put into centrifuge tubes, quickly frozen in liquid nitrogen, and stored at −80 °C for metabolomic analysis. Meanwhile, *G. leucocontextum* was dried at 65 °C for 2–3 days, powdered, and stored for polysaccharide measurement.

### 2.3. Polysaccharide Determination

A standard curve was plotted with the reference substance solution using the sulfuric acid–anthrone method at a wavelength of 625 nm. Then, 2 g of *G. leucocontextum* fruiting-body powder was accurately weighed into a round-bottom flask, 60 mL of water was added, and the mixture was refluxed at a gentle boil for 4 h. The filtrate was obtained by hot filtering. These steps were repeated and the filtrates were combined and concentrated through rotary evaporation at 60 °C to 5 mL. The crude polysaccharides were obtained by adding 75 mL of 95% ethanol dropwise with stirring, standing at 4 °C for 12 h, and centrifuging at 8000 rpm for 10 min. Then, the crude polysaccharides were dissolved in hot water and diluted to 50 mL. The test solutions were obtained by diluting 3 mL of them to 25 mL, and then stored in a 4 °C refrigerator. The absorbances of crude polysaccharides were determined with the same procedure for the standard series and the contents were calculated with the standard curve as follows:

(1) $$X=\frac{c\times V_{1}\times V_{2}}{m\times V_{3}\times 1000}\times 100\%$$

Here, X is the mass of crude polysaccharides in the sample, g/100 g; c is the sugar content in the sample test solution obtained from the standard curve, mg/mL; V_1_ is the first constant volume, 25 mL; V_2_ is the second constant volume, 50 mL; m is the sample mass, g; and V_3_ is the volume of the sample preparation solution transferred during the second constant volume, 3 mL.

### 2.4. Metabolomics

After mixing the stipes and caps of *G. leucocontextum*, they were ground with liquid nitrogen. Then, 100 mg of the powder was transferred into stoppered glass tubes with a 3 mL mixture of chloroform/methanol/water (70/30/0.04, *v*/*v*), and this was then sonicated for 30 min. The extracts were centrifuged at 8000 rpm for 10 min at 4 °C and supernatants were collected. The extraction was repeated and the supernatants were combined and dried under vacuum. The residues were redissolved with 1 mL of methanol and centrifuged at 8000 rpm for 10 min at 4 °C. The testing solutions were obtained by collecting the supernatant and filtering through 0.22 μm membranes [24].

The components were analyzed using an ultra-high-performance liquid chromatography-high resolution mass spectrometry (UPLC-HRMS) system with a Thermo Fisher Hypersil GOLD™ VANQUISH™ C18 UHPLC column (100 mm × 2.1 mm, 1.8 μm). The mobile phase consisted of 0.1% formic acid in water (A) and 0.1% formic acid in acetonitrile (B) for positive-ion mode, and acetonitrile (A) and 5 mM ammonium formate in water (B) for negative-ion mode, with a flow rate at 0.3 mL/min, a column temperature at 25 °C, an injection volume of 3 μL, and a gradient elution program of 90:10 (A/B, *v*/*v*) between 0.0 and 8.0 min, 70:30 (*v*/*v*) between 8.0 and 10.0 min, 67:33 (*v*/*v*) between 10.0 and 13.0 min, 62:38 (*v*/*v*) between 13.0 and 20.0 min, 30:70 (*v*/*v*) between 20.0 and 25.0 min, 5:95 (*v*/*v*) between 25.0 and 28.5 min, and 90:10 (*v*/*v*) between 28.5 and 33 min. Mass spectrometry was performed using an ESI source with a heater temperature of 350 °C, a sheath gas flow rate of 40 arb, an auxiliary gas flow rate of 10 arb, a sweep gas flow rate of 0 arb, a spray voltage of 3.5 kV, a capillary temperature of 320 °C, and a S-lens RF level of 50%. Data were acquired in both positive- and negative-ion modes using a full scan from 150 to 2000 *m*/*z* [23].

### 2.5. Statistical Analysis

Excel was used to plot the polysaccharide standard curve and calculate polysaccharide content [25]. Excel software was employed for data visualization and statistical analysis of polysaccharide data, with *p* < 0.05 or 0.01 being considered significant. For metabolomics, raw data were processed using Compound Discoverer 3.3 to generate a data matrix containing compound names and peak areas [26]. Metabolites were identified using databases such as mzCloud (https://www.mzcloud.org/). Annotation confidence for key compounds (e.g., PGA1) was at MSI levels. Preprocessing steps included filtering missing values, data imputation, normalization, transformation, and scaling. The flavonoid, the diterpenoid and triterpenoids, and the sterol nutrients were extracted from the identified metabolites of the obtained metabolomics. Furthermore, univariate statistical analysis was performed using fold change analysis and Student’s *t*-test, while multivariate analysis utilized principal component analysis (PCA) and orthogonal projections to latent structures discriminant analysis (OPLS-DA). Significantly different metabolites were selected based on variable importance in projection (VIP > 1), fold change (≥2 or ≤0.5), and *p* < 0.05. Pearson correlation analysis was used to identify metabolites associated with polysaccharides. Enrichment analysis of differential metabolites was performed using the KEGG database [27], and metabolic pathway significance was determined via cumulative hypergeometric distribution analysis.

## 3. Results

### 3.1. GMTZ Fruit-Tree Wood–Bagasse Formulation Enhanced Polysaccharides in G. leucocontextum

#### 3.1.1. GMTZ Enhanced the Polysaccharide Content

**Figure 1:**
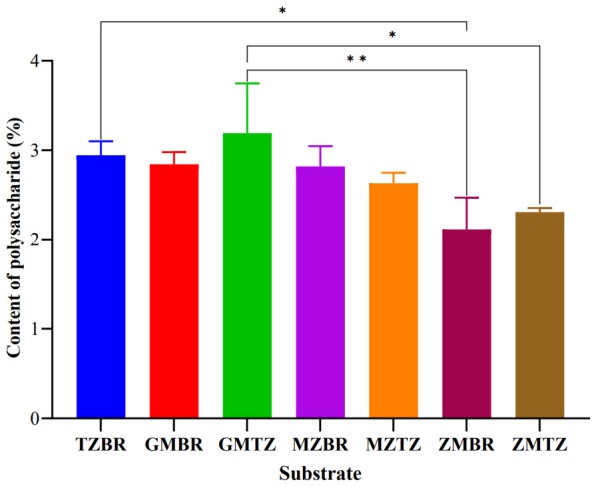
Polysaccharide content of *G. leucocontextum* cultivated with seven substrates of different formulas, including TZBR, ZMBR, ZMTZ, GMBR, GMTZ, MZBR, and MZTZ. * *p* < 0.05; ** *p* < 0.01.

Given that polysaccharides were the significant bioactive substance, the polysaccharide contents of *G. leucocontextum* fruiting bodies cultivated with seven different substrates were determined (Figure 1). At a glance, they showed polysaccharide contents of between 2.11 and 3.19%, in which the fruitbodies grown on GMTZ substrate showed the highest polysaccharide content at 3.19 ± 0.56%, being higher than those of ZMBR (2.11 ± 0.35%, *p* < 0.01) and ZMTZ (2.31 ± 0.05%, *p* < 0.05). This indicates that 30.20% fruit-tree wood and 60.47% bagasse of GMTZ promoted polysaccharide synthesis and accumulation instead of 90.70% oak of ZMBR. Meanwhile, the comparisons between GMTZ and ZMTZ suggested that 30.2% fruit-tree wood would enhance polysaccharide synthesis and accumulation rather than 30.2% oak. TZBR demonstrated the second highest polysaccharide content at 2.95 ± 0.15%, which was higher than ZMBR (*p* < 0.05), indicating that 80% bagasse would increase polysaccharide synthesis and accumulation rather than 90.70% oak. Overall, bagasse and fruit-tree wood may enhance the polysaccharide synthesis and accumulation in the development of *G. leucocontextum*.

#### 3.1.2. GMTZ Appeared a Trade-Off for Most Flavonoids

The flavonoid nutrient profiles of *G. leucocontextum* cultivated on seven distinct formulations were determined using metabolomics (Table 2). However, the fruit-tree wood–bagasse blend (GMTZ) produced the lowest flavonoid content (158.98 ppm), indicating a substrate-dependent trade-off between polysaccharide and flavonoid biosynthesis. The total flavonoid content of other formulations varied significantly, with the cottonseed husk–bagasse mixture (MZTZ) yielding the highest total (613.17 ppm), followed by pure fruit-tree wood (GMBR, 429.52 ppm) and the oak-bagasse mixture (ZMTZ, 401.49 ppm).

Several key flavonoids exhibited distinct substrate preferences. Interestingly, GMTZ specifically promoted naringenin accumulation (8.58 ppm), unlike other substrates. Catechin was highest in pure fruit-tree wood (GMBR, 266.93 ppm), while licochalcone A peaked in MZTZ (223.14 ppm) and ZMTZ (186.94 ppm). Formononetin was notably elevated in cottonseed husk-based substrates, especially MZTZ (37.63 ppm). Oak-rich substrates (ZMBR, ZMTZ) favored syringetin (30.96 and 23.92 ppm) and equol derivatives (equol 4′-O-glucuronide, 31.65 and 46.91), whereas pure cottonseed husk (MZBR) enhanced nobiletin (23.40 ppm). Overall, substrate composition strongly shaped the flavonoid profile.

#### 3.1.3. GMTZ Produced the Most Abundant Diterpenoids and the Lowest Triterpenoid Content

The total content of diterpenoids and triterpenoids in different substrates was calculated (Table 3) and analyzed in conjunction with the substrate formulations. Notably, GMTZ yielded the most abundant diterpenoids (540.06 ppm), characterized by abietic acid (255.59 ppm) and cafestol (218.4 ppm), and the lowest triterpenoid content (752.38 ppm), suggesting that the fruit-tree wood and baggase of GMTZ strongly directed metabolic flux toward diterpenoid synthesis while suppressing most triterpenoids. The highest total triterpenoid content was observed in the cottonseed husk–bagasse mixture (MZTZ, 7891.87 ppm), which was largely driven by an exceptionally high level of glycyrrhetinic acid (7415.91 ppm), followed by the pure bagasse substrate (TZBR, 4833.07 ppm).

#### 3.1.4. GMTZ Led to Specific Enhancement for Androgen Content

The analysis of sterols production in *G. leucocontextum* revealed significant substrate-dependent variations (Table 4). Total sterol content differed markedly among substrates, with the cottonseed husk–bagasse mixture (MZTZ) yielding the highest overall concentration (16,080.74 ppm), nearly double that of the lowest-yielding pure fruit-tree wood substrate (GMBR, 8647.19 ppm). The pure bagasse substrate (TZBR, 12,634.31 ppm) also demonstrated a strong sterol accumulation. Beyond total yield, specific sterol classes exhibited distinct substrate specificity. Notably, the fruit-tree wood–bagasse mixture (GMTZ) led to exceptionally high levels of androgens and related metabolites (6973.35 ppm), driven largely by testosterone propionate and acetate, indicating a specific enhancement of androgen synthesis. In contrast, MZTZ excelled in producing progestogens, vitamin D analogs, and fungal sterols (ergosterol peroxide), while also promoting bile acid conjugates. Pure cottonseed husk (MZBR) favored corticosteroids such as prednisone.

### 3.2. The Metabolomics Unveiled Prostaglandin A1-Phosphoglucomutase for Regulating the Polysaccharides Synthesis in by G. leucocontextum, Hypothetically and Correlatively

#### 3.2.1. PCA Revealed a Profound Impact of the Substrate 

**Figure 2:**
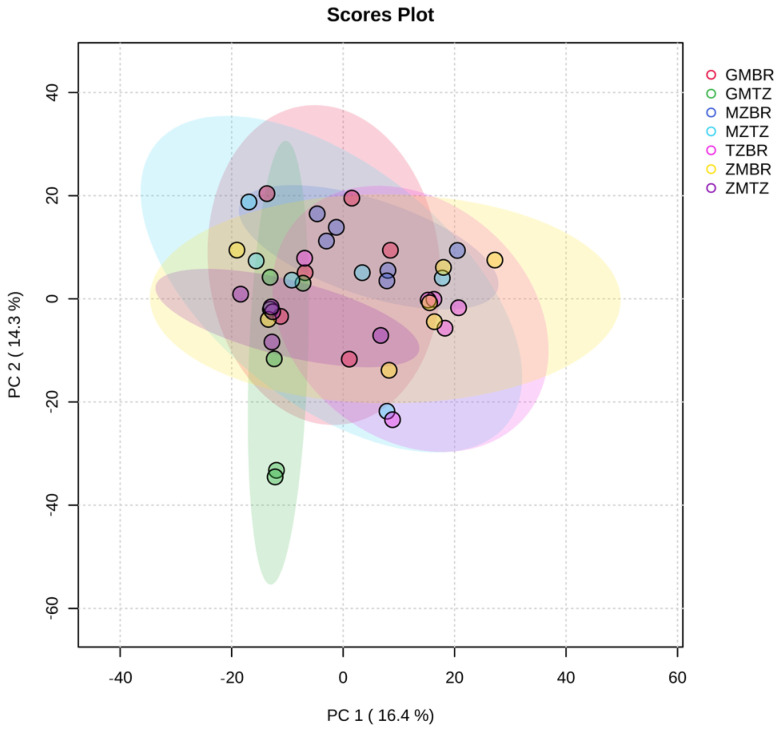
PCA analysis of metabolites of *G. leucocontextum* fruiting bodies under different cultivation formulations.

The PCA plot of the metabolomics data (PC1: 16.4%; PC2: 14.3%) in relation to the seven formulations revealed a clear, structured influence on the metabolic profile of *G. leucocontextum* (Figure 2). The first two principal components collectively explain 30.7% of the total variance, indicating significant metabolic differences between formulations while suggesting that additional sources of variation may be captured in higher components.

According to their main substrate component and mixing strategy, sample groups primarily formed four clusters, including: (1) the fruit-tree wood group (GMTZ, GMBR) in the lower-left quadrant (negative PC1/PC2); (2) the oak group (ZMBR, ZMTZ) in the upper-right (positive PC1/PC2); (3) the non-woody and single-ingredient group (pure cottonseed husk MZBR and pure bagasse TZBR) in the upper-left (negative PC1, positive PC2); and (4) the distinct cottonseed husk–bagasse mixture (MZTZ) in the lower-right quadrant (positive PC1, negative PC2). The tight clustering within the fruit-tree wood and oak groups, regardless of bagasse addition (GMBR *versus* GMTZ), shows that the primary woody component is the dominant driver of metabolic variation. In contrast, the addition of bagasse has a transformative effect only when combined with cottonseed husk, pulling MZTZ into a unique metabolic space separate from both of its individual components (MZBR and TZBR).

#### 3.2.2. GMTZ Showed No Lipid Accumulation and High Hormone Signal, Reinforcing Its Metabolic Shift Toward Polysaccharides and Diterpenoids

**Figure 3:**
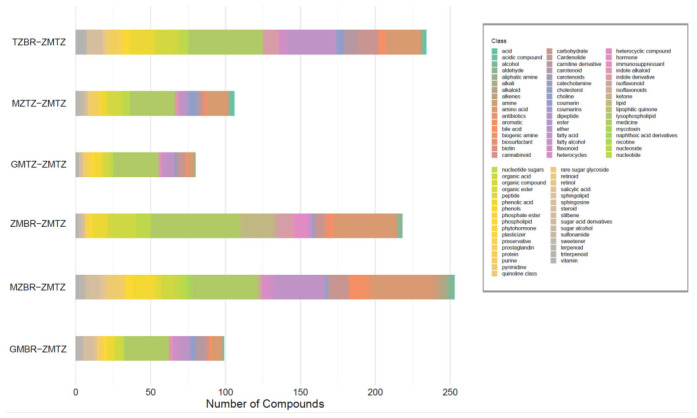
Numbers and types of differential metabolites in *G. leucocontextum* fruiting bodies grown on different substrates.

In ZMTZ–GMTZ (Figure 3), there were 80 metabolites in total, with 36 upregulated and 44 downregulated. Five sugar-related compounds were identified: maltose (4.2866), D-maltose (2.3193), D-glucuronic acid (5.3505), and gluconic acid (2.5692) were upregulated. In ZMTZ–MZTZ, there were 106 metabolites in total, with 59 upregulated and 47 downregulated. Among these, two sugar-related compounds were identified: glucosamine (0.46) was downregulated, and glycogen (5.81) was upregulated. In ZMTZ–ZMBR, a total of 218 differential metabolites were found, including 92 upregulated and 126 downregulated. Seven sugar-related compounds were identified: D-glucosamine was downregulated (0.39), while D-glucosamine-6-phosphate (5.64), maltose (2.28), glycogen (4.24), ribitol (3.89), and L-iditol (5.27) were upregulated. ZMTZ–TZBR had 234 metabolites, with 122 upregulated and 112 downregulated. Fourteen sugar-related compounds were found: 1,5-anhydroglucitol (5.8351), L-iditol (35.016), D-mannose 6-phosphate (2.0827), N-acetylglucosamine (2.1226), glucose (2.128), paracetamol glucuronide (2.1836), N-acetylglucosamine-6-sulfate (2.8006), 1-deoxy-D-xylulose-5-phosphate (3.4798), maltose (3.6091), fructose-6-phosphate (3.9348), glycogen (4.2694), maltose (16.706), and α-lactose (58.256) were upregulated, while neomenthol glucuronide (0.31531) was downregulated. ZMTZ–MZBR had a total of 253 metabolites, with 133 upregulated and 120 downregulated. Twelve sugar-related compounds were found: N-acetyl-D-glucosamine (2.49), N-acetylglucosamine 6-sulfate (2.71), ethyl β-D-glucopyranoside (2.45), maltotriose (5.25), glycogen (10.775), D-(+)-maltose (6.54), D-maltose (3.03), α-lactose (8.09), 1-deoxy-D-xylulose 5-phosphate (3.59), D-ribose 5-phosphate (2.60), D-mannose 6-phosphate (2.35), and D-erythrulose-7-phosphate (2.46) were upregulated. In ZMTZ–GMBR, there were 99 metabolites in total, with 48 upregulated and 51 downregulated. Two sugar-related compounds were identified: menthol glucuronide (0.29) and neomenthol glucuronide (0.29) were downregulated.

**Figure 4:**
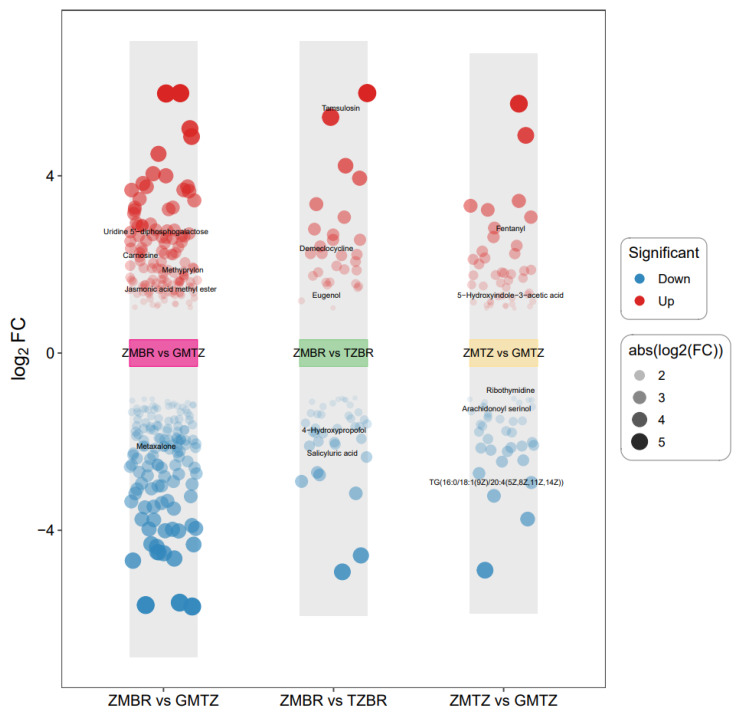
Significance analysis of differential metabolites in *G. leucocontextum* fruiting bodies grown on different substrates.

The volcano plot (Figure 4) reveals distinct metabolic reprogramming in *G. leucocontextum* driven by specific substrate compositions. In the comparison with the pure oak substrate (ZMBR) and the fruit-tree wood–bagasse mixture (GMTZ), ZMBR showed upregulation of metabolites such as uridine analogs, carnosine, and methylprylon, suggesting the induction of stress-responsive or defense-related pathways. In contrast, GMTZ exhibited higher levels of methyl jasmonate, a plant hormone signal, potentially linked to the regulation of secondary metabolism and consistent with its high polysaccharide and diterpenoid yields. When comparing ZMBR to the pure bagasse substrate (TZBR), ZMBR was enriched in arachidonoyl serinol, ribonucleotide analogs, and 4-hydroxyproline derivatives, indicating activation of oxidative stress and membrane lipid remodeling pathways. TZBR, however, accumulated more salicyluric acid, a salicylic acid metabolite, aligning with its enhanced production of fungal sterols like ergosterol peroxide. The comparison between the two woody-bagasse mixtures, such as oak-bagasse (ZMTZ) and fruit-tree wood–bagasse (GMTZ), highlighted the dominant role of the wood type. In detail, ZMTZ specifically upregulated complex triglycerides, pointing to enhanced lipid storage or synthesis, which may synergize with its propensity for androgen-type sterol accumulation, while GMTZ showed no such lipid accumulation, reinforcing its metabolic shift toward polysaccharides and diterpenoids.

#### 3.2.3. Carbohydrate Metabolism and Biosynthesis of Secondary Metabolites Pathways Were Enriched in *G. leucocontextum* Grown on the GMTZ Formulation

**Figure 5:**
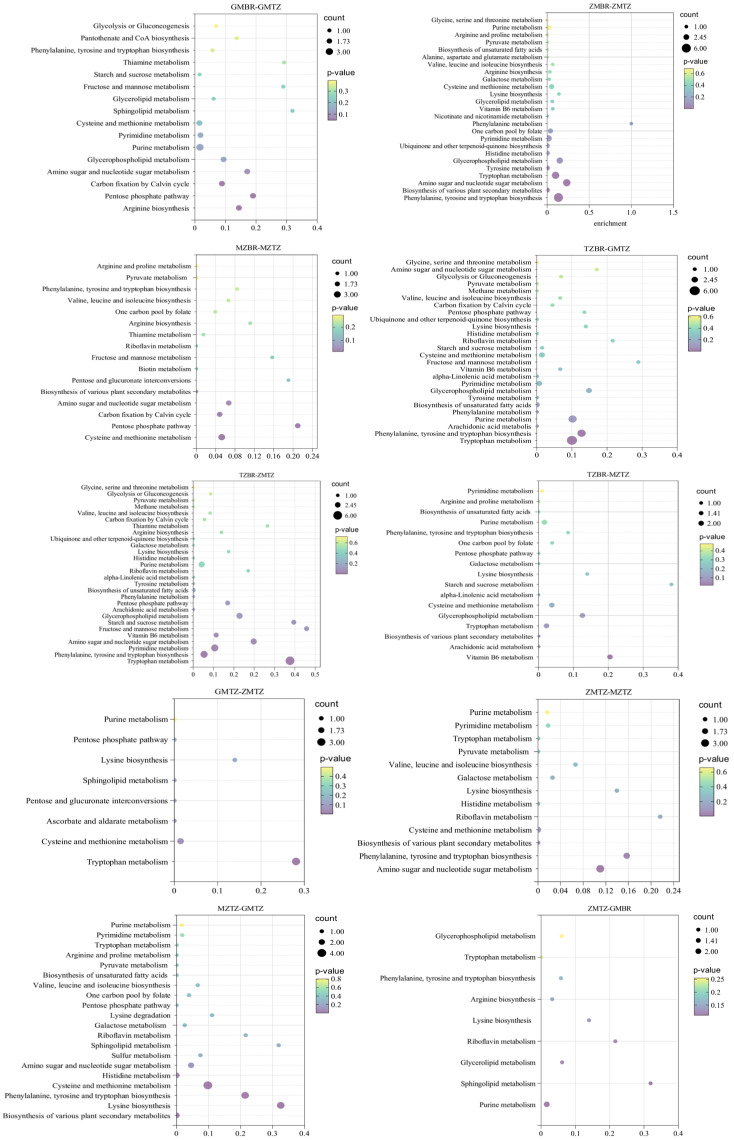
KEGG pathway enrichment analysis of differential metabolites in *G. leucocontextum* fruiting bodies grown on different substrates.

The different metabolites in ZMTZ compared with GMTZ were annotated to 20 metabolic pathways (Figure 5). These included 9 amino acid metabolism pathways, 2 nucleotide metabolism pathways, 4 carbohydrate metabolism pathways, 1 vitamin metabolism pathway, 1 element metabolism pathway, 2 lipid metabolism pathways, and 1 plant secondary metabolism pathway. The significantly enriched metabolic pathways were: biosynthesis of various plant secondary metabolites; lysine biosynthesis; phenylalanine, tyrosine and tryptophan biosynthesis; and cysteine and methionine metabolism. The different metabolites in ZMTZ compared with GMBR were annotated to 25 metabolic pathways. These included 13 amino acid metabolism pathways, 2 nucleotide metabolism pathways, 1 carbohydrate metabolism pathway, 1 terpenoid metabolism pathway, 2 vitamin [28] metabolism pathways, 3 lipid metabolism pathways, and 1 plant secondary metabolism pathway. Five of these metabolic pathways were significantly enriched: phenylalanine, tyrosine and tryptophan biosynthesis; biosynthesis of various plant secondary metabolites; amino sugar and nucleotide sugar metabolism; tryptophan [29] metabolism; and tyrosine metabolism. The different metabolites in ZMTZ compared with TZBR were annotated to 28 metabolic pathways. These included 9 amino acid metabolism pathways, 1 photosynthesis and carbon metabolism pathway, 2 nucleotide metabolism pathways, 1 energy metabolism pathway, 7 carbohydrate metabolism pathways, 1 terpenoid metabolism pathway, 3 vitamin metabolism pathways, and 4 lipid metabolism pathways. Three pathways were significantly enriched: tryptophan metabolism; phenylalanine, tyrosine and tryptophan biosynthesis; and pyrimidine metabolism. The different metabolites in ZMTZ compared with MZTZ were annotated to 13 metabolic pathways. These included 6 amino acid metabolism pathways, 2 nucleotide metabolism pathways, 3 carbohydrate metabolism pathways, and 1 plant secondary metabolism pathway. The significantly enriched metabolic pathways were: amino sugar and nucleotide sugar metabolism; and phenylalanine, tyrosine and tryptophan biosynthesis. The different metabolites in ZMTZ compared with MZBR were annotated to 28 metabolic pathways. These included 9 amino acid metabolism pathways, 5 carbohydrate metabolism pathways, 2 nucleotide metabolism pathways, 3 vitamin metabolism pathways, 7 lipid metabolism pathways, and 2 energy metabolism pathways. Two metabolic pathways were significantly enriched: amino sugar and nucleotide sugar metabolism; and biosynthesis of unsaturated fatty acids.

#### 3.2.4. Correlation Analysis Revealed That Polysaccharide-Rich Substrates Promote a Growth-And-Synthesis Metabolic Phenotype

**Figure 6:**
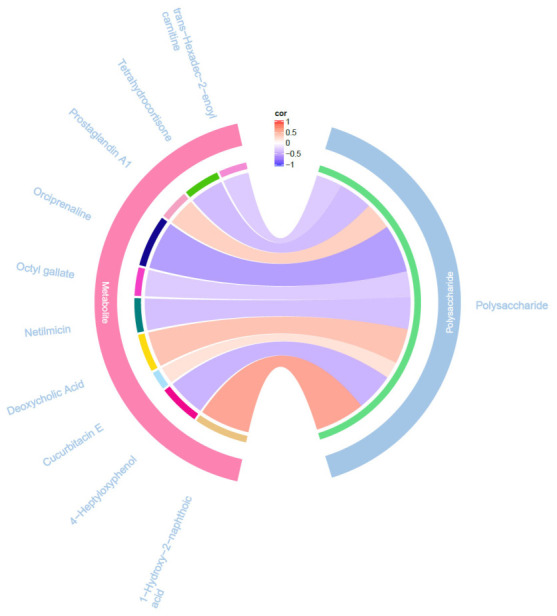
Correlations of polysaccharide contents and metabolites of *G. leucocontextum* fruit bodies under different cultivation substrate formulations.

The correlation analysis (Figure 6) between metabolites and polysaccharide content in *G. leucocontextum* (positive correlations for prostaglandin A1, deoxycholic acid, cucurbitacin E, and 1-hydroxy-2-naphthoic acid; negative correlations for 4-heptyloxyphenol, trans-hexadec-2-enoylcarnitine, tetrahydrocortisone, orciprenaline, octyl gallate, and netilmicin), revealed a fundamental metabolic dichotomy shaped by substrate composition. Polysaccharide-rich substrates, specifically the fruit-tree wood–bagasse mixture (GMTZ, 3.19%), pure bagasse (TZBR, 2.95%), and pure fruit-tree wood (GMBR, 2.84%), promote a growth-and-synthesis metabolic phenotype. These substrates favor the accumulation of positively correlated metabolites, such as the anti-inflammatory and redox-modulating signaling lipid prostaglandin A1 and the triterpenoid cucurbitacin E, whose presence suggests a well-supplied mevalonate pathway and a cellular environment conducive to energy production and biosynthesis. In contrast, polysaccharide-poor substrates, particularly the pure oak substrate (ZMBR, 2.11%) and the oak-bagasse mixture (ZMTZ, 2.31%), induce a “defense-and-stress” metabolic state. These systems accumulate negatively correlated compounds, including xenobiotic detoxification products (the antibiotic analog netilmicin and the bronchodilator analog orciprenaline), lipid stress markers (trans-hexadec-2-enoylcarnitine), and phenolic derivatives (4-heptyloxyphenol). This profile indicates that complex components in oak, such as lignin and tannins, likely trigger chemical defense responses in the fungus, diverting carbon and energy resources away from polysaccharide synthesis.

## 4. Discussion

This study provided a fruit-tree wood–bagasse formulation GMTZ for elevating polysaccharides in *G. leucocontextum* and elucidated the profound, specific influence of its composition on the polysaccharide content [30,31]. Additionally, this targeted metabolic promotion came at the clear cost of the production of most flavonoids [32,33] and triterpenoids [34,35,36], while specifically enhancing androgen synthesis. This specialized profile was reinforced by the absence of significant lipid accumulation [37,38] but higher related hormone signals, indicating a focused metabolic shift.

Substrate composition is a decisive tool for directing the metabolic network of *G. leucocontextum*, yielding distinct bioactive profiles. In detail, the fruit-tree wood–bagasse blend (GMTZ) showed a highly specialized profile, optimally producing polysaccharides [39,40], diterpenoids (abietic acid), and uniquely high levels of androgenic sterols, but at the expense of flavonoids and most other triterpenoids. Pure bagasse (TZBR) served as a robust, balanced substrate supporting polysaccharides, key triterpenoids, and fungal sterols. The cottonseed husk–bagasse mixture (MZTZ) emerged as the most versatile substrate, demonstrating a powerful effect in producing the highest yields of flavonoids, triterpenoids (notably glycyrrhetinic acid), and sterols. A clear metabolic trade-off exists, confirming that no single substrate maximized all compounds. Therefore, cultivation requires aligning the substrate with the target metabolite: GMTZ for immunomodulatory polysaccharides and specific androgens/diterpenoids, TZBR for a balanced profile, and MZTZ for broad-spectrum antioxidants and anti-inflammatory compounds. Future research should elucidate the molecular mechanisms, such as gene expression in the phenylpropanoid and mevalonate pathways, underlying these substrate-induced shifts to enable further optimization.

PCA was a profound tool and confirmed that substrates reshaped the metabolome. PC1 and PC2 components of the PCA analysis accounted for 30.7% of the variance, which may be caused by the highly complex nature of the metabolomes of macrofungi and high biological variation in fruiting bodies. Also, a heavy focus on one species/strain is generally limited by stability. Cultivation on the fruit-tree wood–bagasse substrate (GMTZ) was found to enrich polysacchride and secondary metabolite pathways, promoting a distinct growth-and-synthesis phenotype that prioritizes polysaccharide and diterpenoid production. This clustering strongly aligns with quantitative metabolite data: MZTZ’s (cottonseed husk–bagasse) unique position correlates with its exceptional yield of flavonoids, triterpenoids, and sterols, whereas the fruit-tree wood group specializes in polysaccharides and diterpenoids but not flavonoids. The primary drivers of separation are PC1, associated with wood type (fruit-tree wood *versus* oak), and PC2, linked to the effect of cottonseed husk with bagasse. This PCA map serves as a practical guide for selecting substrates to target specific metabolite classes, such as GMTZ for polysaccharides and androgens and MZTZ for broad-spectrum bioactives, and highlights MZTZ’s potential for developing unique metabolic profiles. Future work should analyze higher principal components and correlate PC scores with key bioactive levels to fully elucidate the underlying mechanisms.

Overall, these metabolic patterns confirmed that the type of woody material, oak *versus* fruit-tree wood, was the primary driver of metabolic divergence, even with bagasse supplementation. Although all samples were harvested at the mature fruiting-body stage, their metabolite dynamics may differ at different phenological stages. Bagasse played a context-dependent role, modifying specific metabolite levels in woody substrates without fundamentally restructuring their core metabolic profile. However, it acted synergistically with cottonseed husk to create a unique metabolome, as exemplified by MZTZ. The differentially abundant metabolites spanned nucleotide modifications, lipid/fatty acid derivatives, plant hormones, amino acid derivatives, and xenobiotic-related compounds, indicating that different substrates activate distinct pathways: fruit-tree wood–bagasse enhanced jasmonate signaling and terpenoid/polysaccharide synthesis; pure bagasse strengthened salicylic acid-mediated resistance and fungal membrane sterol production; and oak-based substrates may trigger oxidative stress and lipid peroxidation. These shifts aligned with bioactive compound data. The elevation of methyl jasmonate in GMTZ supported its high polysaccharide/diterpenoid phenotype, while increased salicyluric acid in TZBR aligned with its fungal sterol production. The upregulation of nucleotide/lipid derivatives in oak substrates (ZMBR/ZMTZ) corresponded to higher yields of certain triterpenoids and androgenic steroids, possibly sharing the mevalonate pathway. Consequently, substrate selection must match the target metabolite class: fruit-tree wood–bagasse (GMTZ) for polysaccharides and hormone-associated pathways; cottonseed husk–bagasse (MZTZ) for broad-spectrum bioactive combinations; and oak-based systems for lipid-related or stress-induced products.

Functionally, metabolites positively correlated with polysaccharides may support synthesis by maintaining redox homeostasis (prostaglandin A1), aiding energy generation (deoxycholic acid), indicating precursor abundance (cucurbitacin E), or reflecting efficient substrate use (1-hydroxy-2-naphthoic acid). Among these, prostaglandin A1 is a metabolite identified by metabolomic analysis. Its concurrence has not been reported for *Ganoderma species* and its function in fungi remains unclear. Conversely, negatively correlated metabolites signaled inhibitory processes like fatty acid oxidation stress (trans-hexadec-2-enoylcarnitine), physiological stress (tetrahydrocortisone), resource-intensive detoxification (orciprenaline, netilmicin), or enzyme inhibition (alkylphenols). To optimize polysaccharide yield, substrates must promote this “growth-and-synthesis” phenotype while minimizing defense-and-stress responses. Fruit-tree wood and bagasse GMTZ were preferred, as they enhanced positive correlates and suppressed negative ones, whereas oak potently induced the defense metabolism and should be avoided. The cottonseed husk–bagasse mixture (MZTZ) represented a unique case, balancing positive correlates with a broader bioactive profile rather than specializing in polysaccharides. These confirmed that efficient polysaccharide synthesis requires unimpeded energy metabolism and minimal defense burden, which was a state best achieved with fruit-tree wood and bagasse substrates. Subsequent research should quantify these biomarkers, perform pathway perturbation, and use multi-omics integration to identify key regulatory genes.

**Figure 7:**
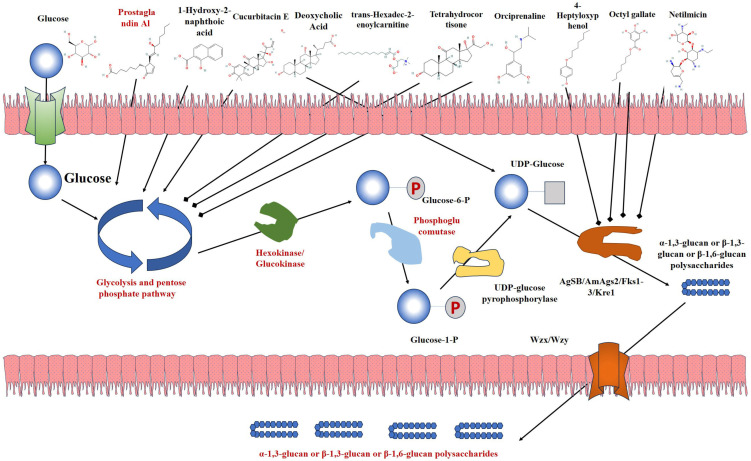
The hypothetical and correlation-based prostaglandin A1-phosphoglucomutase mechanism for enhancing polysaccharide synthesis by *G. leucocontextum*. The red colored symbols were the key components in the proposed mechanism. Arrow meant positive regulation and dot meant negative regulation.

The hypothetical and correlation-based prostaglandin A1-phosphoglucomutase was proposed as a mechanism for enhancing polysaccharide synthesis by *G. leucocontextum* (Figure 7). Carbon sources like cellulose in ZMTZ were degraded into glucose and then metabolized through glycolysis and the pentose phosphate pathway, generating various sugar phosphates, including glucose-6-phosphate and fructose-6-phosphate [2,41,42]. A key step is the conversion of sugar phosphates into uridine diphosphate-sugars (UDP-sugars), such as UDP-glucose. These serve as the direct, activated precursors for polysaccharide chain elongation [43]. The activated monosaccharides are sequentially linked into polysaccharides, catalyzed by polysaccharide synthase complexes, such as AgSB, AmAgs2, Fks1-3, and Kre1. The resulting polysaccharide chains are subsequently transported extracellularly by Wzx/Wzy or integrated into cellular structures.

Among these, prostaglandin A1 is a prostaglandin, which may act as a signaling molecule that activates cellular stress or defense response pathways. This may indirectly upregulate the expression of genes related to polysaccharide synthesis or promote precursor transport by influencing membrane fluidity. 1-Hydroxy-2-naphthoic acid is an aromatic compound, which may serve as a cofactor or precursor involved in the synthesis of quinones or other secondary metabolites. These metabolites might, in turn, stabilize enzyme complexes or act as electron carriers, promoting sugar metabolic flux. Deoxycholic Acid is a bile acid, whose primary theoretical role is to enhance cell membrane fluidity. A more fluid membrane facilitates the transmembrane transport of precursors like UDP-sugars to the synthesis sites, thereby improving polymerization efficiency. Cucurbitacin E is a triterpenoid defense compound, whose presence typically indicates that the cell is under mild biotic stress. This stress may trigger a general fungal stress response, which includes accelerating the synthesis of protective polysaccharides to reinforce the cell wall, such as β-glucans. Both 4-heptyloxyphenol and octyl gallate are phenolic antioxidants. The polymerization and cross-linking of polysaccharide chains sometimes require specific oxidative steps. Excess exogenous antioxidants may disrupt this endogenous redox balance, thereby inhibiting polymerization. Trans-hexadec-2-enoylcarnitine is an acylcarnitine form of a long-chain fatty acid and its accumulation indicates active fatty acid β-oxidation. This process heavily consumes coenzyme A and generates acetyl-CoA, potentially competing with glycolysis for cofactors and carbon flux, thus depriving polysaccharide synthesis of necessary energy and precursors. Tetrahydrocortisone is a steroid hormone metabolite. Exogenous steroids may feedback-inhibit or interfere with the fungus own sterol metabolism. Since the sterol metabolism is closely linked to cell membrane integrity and signaling, this could ultimately inhibit primary metabolism and growth, reducing polysaccharide synthesis. Orciprenaline is a β-adrenergic receptor agonist, but its direct impact on fungi is not fully understood. One hypothesis is that it might mimic a certain stress signal, mistakenly redirecting cellular resources away from anabolic processes like polysaccharide synthesis to other pathways. Netilmicin is an aminoglycoside [44] antibiotic whose classic role is to inhibit prokaryotic protein synthesis. In fungi, it may exert similar toxicity, inhibiting mitochondrial function or the synthesis of key enzymatic proteins, directly poisoning the cell and leading to a shutdown of the global metabolism, including polysaccharide synthesis.

This integrated model indicates that polysaccharide synthesis is a comprehensive process finely regulated by energy status, precursor supply, redox balance, and external signals. The biomarkers identified were mapped to different nodes within this network from various angles. Positively correlated compounds mostly promote polysaccharide accumulation by providing support, enhancing transport, or inducing protective synthesis responses. Negatively correlated compounds inhibit synthesis by consuming competing resources, introducing toxicity, or disrupting key signals. Among these, the prostaglandin A1-phosphoglucomutase was hypothetically proposed to be involved in enhancing polysaccharide synthesis in *G. leucocontextum*.

## 5. Conclusions

In summary, this study investigated the impact of substrate formulations on the active polysaccharide content and on metabolomic changes in *G. leucocontextum*. The fruit-tree wood–bagasse formulation GMTZ (30.20% fruit-tree wood, 60.47% bagasse) yielded fruiting bodies with the highest polysaccharide content (3.19 ± 0.56%). Metabolomic analysis revealed that GMTZ functions as a specialized substrate, efficiently channeling resources toward the biosynthesis of polysaccharides and diterpenoids at the expense of flavonoids and most triterpenoids. This growth-and-synthesis metabolic phenotype, characterized by enriched polysaccharide and secondary metabolite pathways, was unique and was further associated with a dramatic, specific enhancement of androgen synthesis. Furthermore, PCA confirmed that substrate composition reshaped the metabolomics profoundly, demonstrating that GMTZ promoted growth-and-synthesis metabolic phenotype and lignin-rich formulations induced a defense-and-stress phenotype. Moreover, prostaglandin A1, deoxycholic acid, cucurbitacin E, and 1-hydroxy-2-naphthoic acid were found to be positively correlated with polysaccharide synthesis. In addition, networks for polysaccharide biosynthesis were mapped and it was proposed that prostaglandin A1-phosphoglucomutase may be a mechanism for enhancing polysaccharides by GMTZ. This research provided a substrate formulation for elevating polysaccharides in *G. leucocontextum*.

## Figures and Tables

**Table 1:** Seven formulations for *G. leucocontextum* cultivation and their abbreviations.

No.	Substrate	Formula (by Mass Weight Ration)	Charecteristics
1	TZBR	80.00% bagasse, 12.00% wheat bran, 5.00% soybean meal, 1.50% calcium carbonate, 1.50% calcium powder	80.00% bagasse
2	ZMBR	90.70% oak, 5.00% wheat bran, 3.00% soybean meal, 0.60% calcium carbonate, 0.60% calcium powder	90.70% oak
3	ZMTZ	30.20% oak, 60.47% bagasse, 5.00% wheat bran, 3.00% soybean meal, 0.60% calcium carbonate, 0.60% calcium powder	30.20% oak and 60.47% bagasse
4	GMBR	90.70% fruit-tree wood, 5.00% wheat bran, 3.00% soybean meal, 0.60% calcium carbonate, 0.60% calcium powder	90.70% fruit-tree wood
5	GMTZ	30.20% fruit-tree wood, 60.47% bagasse, wheat bran 5.00%, 3.00% soybean meal, 0.60% calcium carbonate, 0.60% calcium powder	30.20% fruit-tree wood, 60.47% bagasse
6	ZMBR	86.70% cottonseed husk, 7.20% wheat bran, 4.33% soybean meal, 0.87% calcium carbonate, 0.87% calcium powder	86.70% cottonseed husk
7	MZTZ	43.35% cottonseed husk, 43.35% bagasse, 7.20% wheat bran, 4.33% soybean meal, 0.87% calcium carbonate, 0.87% calcium powder	43.35% cottonseed husk, 43.35% bagasse

**Table 2:** The landscape of the flavone nutrients (ppm) of *G. leucocontextum* cultivated with different substrates.

Compound	Content (ppm)						
	TZBR	GMBR	GMTZ	ZMBR	ZMTZ	MZBR	MZTZ
2-(2-Amino-3-methoxyphenyl)-4H-1-benzopyran-4-one	1.01	1.07	0.71	2.37	1.28	1.71	1.70
2′,4′-Dihydroxy-6′-methoxy-3′-methylchalcone	5.93	5.71	3.59	5.90	7.78	10.86	9.22
3′-O-methyl-(-)-epicatechin	20.91	2.57	2.21	12.38	2.33	3.51	3.91
4′-O-methyl-(-)-epicatechin-3′-O-beta-Glucuronide	4.65	13.28	4.17	6.89	12.42	22.04	20.36
Catechin	25.23	266.93	77.32	44.80	72.75	63.44	179.27
Dihydrodaidzein	5.55	0.82	2.58	0.96	2.16	0.46	4.89
Equol	11.49	5.04	2.77	22.37	5.20	3.69	6.96
Equol 4′-O-glucuronide	24.81	13.75	5.39	31.65	21.52	5.99	46.91
Eriodictyol	2.76	1.12	0.81	5.23	0.88	1.76	1.12
Formononetin	16.31	6.52	5.86	4.88	8.83	18.83	37.63
Irisolidone 7-O-glucuronide	5.97	9.33	3.92	5.54	4.26	3.30	6.82
Licochalcone A	108.80	35.58	9.19	77.39	186.94	3.97	223.14
Naringenin	0.74	1.16	8.58	1.48	1.52	0.58	1.39
Nobiletin	8.65	13.24	12.21	9.64	15.49	23.40	11.29
O-Desmethylangolensin	12.37	9.89	4.75	13.81	4.50	9.41	12.46
Phloretin 2′-O-glucuronide	18.66	20.80	8.97	29.93	29.71	7.67	29.02
Syringetin	13.81	22.71	5.95	30.96	23.92	5.51	17.08
Flavone nutrients total	287.65	429.52	158.98	306.18	401.49	186.13	613.17

**Table 3:** The landscape of the diterpenoid and triterpenoids nutrients of *Ganoderma leucocontextum*, grown by different substrates.

Class	Compound	Content (ppm)						
		TZBR	GMBR	GMTZ	ZMBR	ZMTZ	MZBR	MZTZ
Diterpenoids	10-Deacetylbaccatin III	43.89	28.18	40.98	22.25	27.69	5.27	36.81
	Abietic acid	90.28	71.88	255.59	43.81	93.6	29.13	49.25
	Andrographolide	22.19	75.39	18.53	14.33	30.8	36.8	67.48
	Cafestol	191.7	142.7	218.4	143.34	50.3	9.77	24.58
	Forskolin	12.74	25.07	6.56	31.17	57.11	52.37	63.98
	Total diterpenoids	360.8	343.22	540.06	254.9	259.5	133.34	242.1
Triterpenoids	Cucurbitacin E	12.43	4.87	4.51	7.49	92.52	14.44	37.15
	Cucurbitacin I	14.99	6.57	3.93	22.3	24.41	10.66	10.44
	Cucurbitacin S	205.7	103.87	161.81	133	162.14	39.28	113.67
	Cyclopamine	22.56	48.37	30.57	84.05	85.61	30.2	61.8
	Diosgenin	89.79	14.52	9.44	22.4	17.32	31.97	146.78
	Glycyrrhetinic acid	4400.81	933.84	485.74	1928.71	994.16	1803.13	7415.91
	Ruscogenin	86.79	50.8	56.38	23.33	66.98	47.88	106.12
	Total triterpenoids	4833.07	1162.84	752.38	2221.28	1443.14	1977.56	7891.87
	Total terpenoids	5193.87	1506.06	1292.44	2476.18	1702.64	2110.9	8133.97

**Table 4:** The landscape of the sterol nutrients of *G. leucocontextum*, grown by different substrates.

Classes	Compound	Different Substrates						
		TZBR	GMBR	GMTZ	ZMBR	ZMTZ	MZBR	MZTZ
Primary sterols of membranes	7-Ketocholesterol	56.14	24.04	15.40	21.50	23.51	9.75	11.51
	Cholesterol sulfate	44.74	57.18	72.92	45.74	44.83	33.16	23.79
Total primary sterols of membranes		100.88	81.22	88.32	67.24	68.34	42.91	35.30
Vitamin D and analogs	Ergocalciferol	8.92	21.19	2.67	5.87	4.40	148.52	215.08
	25-Hydroxyvitamin D2	15.13	14.6	6.31	26.57	10.85	20.36	51.66
	Calcipotriol	9.56	23.77	8.23	11.86	43.81	53.66	46.01
	24,25-Dihydroxyvitamin D	57.99	13.32	3.52	3.22	31.17	8.12	34.08
Total vitamin D and analogs		91.60	72.88	20.73	47.52	90.23	230.66	346.83
Fungal sterols	Ergosterol peroxide	951.08	294.96	207.38	494.5	298.88	388.45	718.59
Plant sterols	beta-Vatirenene	83.74	15.75	5.61	9.12	8.16	6.21	16.4
Androgens and metabolites	Androstenedione	159.03	107.61	64.78	107.24	98.68	110.97	227.58
	Androsterone	7.58	8.45	263.94	7.92	9.30	4.64	12.57
	Androsterone sulfate	0.71	0.95	16.08	0.79	0.98	0.52	1.31
	Testosterone acetate	57.4	20.15	2424.47	45.41	13.24	6.25	17.21
	Testosterone cypionate	336.01	247.34	54.39	175.87	121.26	247.10	421.57
	Testosterone enanthate	369.77	168.50	74.39	286.61	111.33	195.88	393.23
	Testosterone propionate	24.13	25.23	3034.05	27.45	24.57	11.46	31.91
	Testosterone undecanoate	1428.24	294.50	183.49	659.42	364.36	660.83	2566.69
	5α-Dihydrotestosterone	6.45	13.61	17.60	5.74	26.24	4.68	55.09
	19-Norandrosterone	116.03	234.72	34.72	97.7	186.18	119.67	244.33
	Nandrolone	43.07	70.08	27.25	42.15	53.81	40.78	81.41
	Nandrolone decanoate	17.87	6.66	45.19	12.52	12.47	4.00	10.19
	11-Hydroxyandrosterone	15.05	17.79	150.29	14.34	17.31	9.52	32.62
	11β-Hydroxyandrosterone	254.10	241.80	189.06	211.55	215.58	278.23	522.89
	Fluoxymesterone	386.77	463.42	245.01	329.14	360.34	199.61	576.19
Total androgens and metabolites		3330.27	2024.75	6973.35	1830.93	1588.68	1928.88	5078.48
Progestogens and metabolites	Progesterone	229.28	129.70	14.00	349.37	47.84	116.44	159.23
	17-Hydroxyprogesterone	57.32	102.94	47.2	75.94	57.33	90.15	137.16
	21-Hydroxypregnenolone	3770.59	2374.49	1231.29	2189.14	2934.54	2990.65	4082.36
	Pregnenolone	106.04	103.51	53.46	106.45	102.51	75.78	106.04
	16-a-Hydroxypregnenolone	1516.65	1275.97	631.83	1064.48	1212.33	1712.86	2067.65
	Allopregnanolone	18.74	18.45	49.21	19.36	49.42	10.75	25.87
	Cortexolone	5.73	5.05	12.05	4.17	9.49	9.79	26.03
	Desogestrel	11.67	15.76	12.23	16.74	16.24	28.96	24.29
	2-Hydroxydesogestrel	322.80	458.12	207.47	187.5	174.6	203.90	308.27
	Drospirenone	6.74	9.07	4.21	2.89	2.54	12.42	15.97
	Levonorgestrel	8.36	2.97	3.82	19.16	17.85	4.70	23.94
	Norethindrone	5.53	9.66	4.52	2.36	3.35	3.58	5.83
	15-beta-hydroxycyproterone acetate	6.8	45.22	5.96	15.08	23.89	7.00	17.67
Total progestogens and metabolites		6356.42	4950.59	2326.61	4268.22	4754.88	5337.81	6726.37
Corticosteroids and metabolites	Aldosterone	0.96	1.64	0.79	0.99	2.29	3.31	1.85
	Tetrahydrocorticosterone	68.74	95.53	54.76	60.36	216.84	92.00	158.18
	Tetrahydrocortisone	9.59	8.66	30.71	6.61	8.63	5.61	16.00
	Tetrahydrodeoxycorticosterone	137.29	191.41	93.94	175.74	216.34	79.98	318.00
	Tetrahydrodeoxycortisol	68.20	75.18	17.54	29.91	55.02	46.85	78.04
	5alpha-Tetrahydrocortisol	5.55	11.50	8.69	16.39	8.28	11.51	21.85
	18-Oxocortisol	8.65	1.48	0.98	4.16	3.47	5.84	16.48
	11-Dehydrocorticosterone	6.41	1.42	0.92	2.33	3.69	2.00	4.67
	Cortexolone	5.73	5.05	12.05	4.17	9.49	9.79	26.03
	Deoxycorticosterone	49.35	8.46	35.91	32.24	20.17	4.25	7.34
	Fludrocortisone	10.80	10.59	106.96	9.49	11.56	7.95	15.93
	Methylprednisolone	59.15	6.72	4.99	6.4	7.56	4.39	10.12
	Prednisone	248.78	62.21	39.91	397.79	183.60	1004.14	627.64
Total corticosteroids and metabolites		668.57	475.61	369.52	735.4	737.17	1426.31	1556.59
Adrenal androgens	Dehydroepiandrosterone	5.81	7.14	5.2	4.10	6.07	5.39	8.84
	16-alpha-Hydroxy DHEA 3-sulfate	22.86	69.43	16.51	16.22	95.08	36.55	93.98
Total adrenal androgens		28.67	76.57	21.71	20.32	101.15	41.94	102.82
Primary and secondary bile acids	Deoxycholic Acid	4.87	2.66	1.68	15.15	10.88	1.66	13.18
	Lithocholic acid glycine conjugate	109.78	5.54	5.06	21.12	6.03	5.15	8.07
	Varanic acid	164.16	39.54	16.32	64.24	32.58	27.4	54.72
	3alpha,7alpha-Dihydroxycoprostanic acid	19.57	5.93	2.06	2.55	13.27	3.02	13.36
	3-beta,7-alpha-Dihydroxy-5-cholestenoate	28.96	10.71	2.69	15.78	7.29	23.56	14.88
	3-beta,7-alpha-Dihydroxychol-5-en-24-oic Acid	9.37	10.89	30.83	9.81	10.25	3.00	27.55
Total primary and secondary bile acids		291.71	74.27	57.68	119.65	80.09	60.78	131.76
Bile acid conjugates	Deoxycholic acid glycine conjugate	181.88	145.98	185.80	54.03	446.29	469.31	361.87
	Glycocholic acid	4.95	12.93	7.82	4.85	17.17	8.07	33.35
	Taurocholic acid	95.06	42.83	42.19	58.85	57.46	19.86	93.37
	Lithocholyltaurine	104.48	99.11	40.75	83.37	84.12	165.46	192.42
	3-Sulfodeoxycholic acid	168.42	311.42	320.38	446.56	364.51	252.69	382.69
Total bile acid conjugates		554.79	610.27	590.94	647.66	969.55	915.39	1063.7
Steroidal sapogenins	Diosgenin	89.79	14.52	9.44	22.4	17.32	31.97	146.78
	Ruscogenin	86.79	50.8	56.38	23.33	66.98	47.88	106.12
Total steroidal sapogenins		176.58	65.32	65.82	45.73	84.30	79.85	252.90
Total sterol nutrients		12,634.31	8647.19	10,727.67	8887.29	8780.23	10,458.4	16,080.74

## Data Availability

No new data were created or analyzed in this study. Data sharing is not applicable to this article.
